# Light Intensity Physical Activity and Sedentary Behavior in Relation to Body Mass Index and Grip Strength in Older Adults: Cross-Sectional Findings from the Lifestyle Interventions and Independence for Elders (LIFE) Study

**DOI:** 10.1371/journal.pone.0116058

**Published:** 2015-02-03

**Authors:** David Bann, Don Hire, Todd Manini, Rachel Cooper, Anda Botoseneanu, Mary M. McDermott, Marco Pahor, Nancy W. Glynn, Roger Fielding, Abby C. King, Timothy Church, Walter T. Ambrosius, Thomas Gill, for the LIFE Study Group

**Affiliations:** 1 MRC Unit for Lifelong Health and Ageing at UCL, London, United Kingdom; 2 Department of Biostatistical Sciences, Division of Public Health Sciences, Wake Forest School of Medicine, Winston-Salem, North Carolina, United States of America; 3 Department of Aging & Geriatric Research, University of Florida, Gainesville, Florida, United States of America; 4 Department of Health Policy Studies and Institute of Gerontology, University of Michigan—Dearborn/Ann Arbor, Ann Arbor, Michigan, United States of America; 5 Northwestern University, Feinberg School of Medicine, Chicago, United States of America; 6 Department of Epidemiology, University of Pittsburgh, Pittsburgh, United States of America; 7 Nutrition, Exercise Physiology, and Sarcopenia Laboratory, Jean Mayer USDA Human Nutrition Research Center on Aging at Tufts University, Boston, MA, United States of America; 8 Stanford University School of Medicine, Stanford, CA, United States of America; 9 Pennington Biomedical Research Center, Baton Rouge, Louisiana, United States of America; 10 Yale School of Medicine, New Haven, Connecticut, United States of America; San Francisco Coordinating Center, UNITED STATES

## Abstract

**Background:**

Identifying modifiable determinants of fat mass and muscle strength in older adults is important given their impact on physical functioning and health. Light intensity physical activity and sedentary behavior are potential determinants, but their relations to these outcomes are poorly understood. We evaluated associations of light intensity physical activity and sedentary time—assessed both objectively and by self-report—with body mass index (BMI) and grip strength in a large sample of older adults.

**Methods:**

We used cross-sectional baseline data from 1130 participants of the Lifestyle Interventions and Independence for Elders (LIFE) study, a community-dwelling sample of relatively sedentary older adults (70-89 years) at heightened risk of mobility disability. Time spent sedentary and in light intensity activity were assessed using an accelerometer worn for 3–7 days (Actigraph GT3X) and by self-report. Associations between these exposures and measured BMI and grip strength were evaluated using linear regression.

**Results:**

Greater time spent in light intensity activity and lower sedentary times were both associated with lower BMI. This was evident using objective measures of lower-light intensity, and both objective and self-reported measures of higher-light intensity activity. Time spent watching television was positively associated with BMI, while reading and computer use were not. Greater time spent in higher but not lower intensities of light activity (assessed objectively) was associated with greater grip strength in men but not women, while neither objectively assessed nor self-reported sedentary time was associated with grip strength.

**Conclusions:**

In this cross-sectional study, greater time spent in light intensity activity and lower sedentary times were associated with lower BMI. These results are consistent with the hypothesis that replacing sedentary activities with light intensity activities could lead to lower BMI levels and obesity prevalence among the population of older adults. However, longitudinal and experimental studies are needed to strengthen causal inferences.

## Background

Obesity is highly prevalent and is associated with impaired physical functioning,[1,2] morbidity, and premature mortality.[3] In addition, low muscle strength is associated with greater physical impairment[1,4] and higher mortality rates.[5] Obesity and low muscle strength have been found to commonly co-occur among older adults—this syndrome, sometimes referred to as dynapenic obesity, has been associated with even higher risk of impaired functioning and disability than either obesity or muscle weakness alone.[6–8] Fat mass and muscle strength are therefore two important outcomes that may independently or synergistically affect physical functioning and health.[9] As such, there is a need to identify factors that are associated with measures of fat mass and muscle strength, especially those that may be modifiable such as physical activity and sedentary behaviours.

Intervention studies of older adults have found that moderate to vigorous intensity exercise leads to short-term losses in weight,[10–12] and resistance exercise results in increased muscle strength.[13] Observational studies can add complementary information on how free-living activities of lower intensities (including non-volitional activities) relate to fat mass and muscle strength, although few have been conducted in older adults. While some studies have found that higher physical activity is associated with lower fat mass[14] or body mass index (BMI) and stronger grip in older adults,[15–17] others have produced equivocal findings.[18–21] For example, in a large European study (N = 288,498), higher self-reported physical activity was associated with lower weight gain in older women but not older men.[20] Most existing studies have used self-reported measures of total or moderate to vigorous physical activity and have not examined light intensity activity, which older adults frequently undertake but find difficult to recall.[22,23] Light intensity activities, such as leisurely walking or light housework, contribute to energy expenditure[24] and may therefore contribute to lowering fat mass levels by improving energy balance (the ratio of energy expenditure to intake). Light intensity activities are likely to be especially important among older adults as they are at greater risk of falls and health-related problems, which may preclude participation in activities of higher intensity.

Increasingly, sedentary behavior has been associated with a wide range of health outcomes independent of physical activity level.[25,26] Greater sedentary time has also been related to indicators of higher fat mass in some[14,27] but not all studies,[28] and to weaker grip strength.[29] Most studies, however, have used only self-reported measures of sedentary time and few have been conducted in older adults. As older adults typically spend substantial proportions of their day sedentary (~60% of waking hours),[30] it is important to understand how sedentary time relates to indicators of fat mass and muscle strength. As the effect of sedentary time on outcomes may differ by the type of sedentary behavior undertaken (eg, television viewing vs computer use[29]), analyzing different types of behavior may be useful to help identify the most relevant intervention targets.

The objective of this study was to evaluate the associations of sedentary time and light intensity physical activity with BMI and grip strength among older men and women using baseline data from the Lifestyle Interventions and Independence for Elders (LIFE) Study. This study benefits from the availability of measures of both objectively assessed and self-reported data on physical activity and sedentary behavior in older adults.

## Methods

### Study population

The LIFE Study is a Phase 3 randomized clinical trial, conducted at 8 field centers in 8 US states, that is designed to compare a long-term physical activity program with a health education program on the incidence of major mobility disability (clinicaltrials.gov Identifier: NCT01072500). The rationale, design, and methods of the LIFE Study have been presented in detail elsewhere,[31] as have baseline characteristics.[32] Participants aged 70–89 years were eligible if they were at heightened risk of mobility disability (i.e., a Short Physical Performance Battery score ≤9), yet were able to walk 400m in 15 minutes without sitting, leaning, or assistance from a walker or another person. Participants were additionally required to be relatively sedentary (i.e., reported <20 mins/week in the past month getting regular physical activity and ≤125 mins/week of moderate to vigorous activity). In total, 1635 participants (~67% women) were enrolled—ranging from 200 to 216 per site. The study was given ethical approval by the institutional review boards at all participating sites (University of Florida, Northwestern University, Pennington Biomedical Research Center, University of Pittsburgh, Stanford University, Tufts University, Wake Forest University, and Yale University), and written informed consent was obtained from all study participants.

### Body mass index and grip strength assessment

Weight, height, and waist circumference were measured by research staff using a standard protocol, and BMI (kg/m^2^) was derived. Hand grip strength (kg) was assessed twice consecutively in the dominant arm using a handheld Jamar dynamometer, or in the non-dominant hand if participants reported surgery or a current flare-up of pain in the dominant hand/wrist in the past 3 months (N = 46). The maximum value was used in analyses.

### Physical activity and sedentary behavior measures

**Objectively assessed measures**. Participants were asked to wear an accelerometer (Actigraph GT3X) on their hip for seven consecutive days except during sleep, showering/bathing, and swimming. Movement was captured along the vertical axis in 1-minute epochs, and non-wear time was defined as 90 minutes of consecutive zero counts as described by Choi and colleages.[33] In total, 1296 participants had valid baseline accelerometer data. Missing participant data were largely due to technical or procedural difficulties during the data collection phase. All analyses were constrained to participants who wore the device for 600 minutes per day for three or more days (34 participants were excluded due to low wear time); the total analytic sample (excluding participants with missing covariate or outcome data) was 1193 for BMI, and 1130 for grip strength (73% and 69% of the baseline sample, respectively).

A measure of total physical activity was derived by calculating the daily average number of counts per minute. This has been shown to be positively correlated with physical activity energy expenditure assessed using oxygen consumption in older adults.[34] In the absence of well-accepted, evidence-based cut-points for sedentary behavior and physical activity intensity in older adults,[35] cut-points were chosen that demarcate sedentary time (<100 counts/minute) and time spent in light intensity activity. As previously described, light intensity activity was categorized as time spent in lower-light (100–1040 counts/minute) and higher-light activity (1041–1951 counts/minute), as evidence suggests that spending more time in higher-light activity may be particularly beneficial for health outcomes in older adults.[34,36] Time spent in moderate to vigorous activity was not used as a main exposure as it was expected that screening of the LIFE Study sample[31] would result in low levels of and little variation in moderate to vigorous activity. Data for all categories were expressed in minutes or hours/day, and all data were adjusted for wear time by its inclusion as a covariate in models.

**Self-reported measures**. During the baseline assessment, participants completed the Community Healthy Activities Model Program for Seniors physical activity questionnaire (CHAMPS).[37] This instrument measured the time spent undertaking 41 domestic and leisure time activities in a typical week during the past 4 weeks. Activities were grouped according to the estimated energy cost of each activity—i.e., time spent sedentary (≤1.5 metabolic equivalents [METs]),[38] and in lower-light (>1.5–2 METs) and higher-light intensity activity (>2 METs).[24] As previously described,[39] where CHAMPs items comprised multiple activities, an average MET value was assigned. The questionnaire items used in each category are shown in S1 Appendix. Three separate components of sedentary time which were expected to be commonly undertaken in old age (television viewing, computer use, and reading) were also used as separate exposures in analyses to examine whether they were differentially associated with BMI and grip strength. All self-reported measures were converted so that resulting regression coefficients showed the change in outcome per hour/day increase in activity.

### Potential confounders

The following factors were considered as potential confounders because they have previously been shown to be associated with both the exposures and outcomes of interest: age, sex, ethnicity, education, clinical site, smoking, alcohol consumption, number of chronic conditions (congestive heart failure, myocardial infarction/angina, peripheral arterial disease, stroke, cancer), history of arthritis or rheumatism, and self-rated health.

### Analytical strategy

Descriptive statistics (mean (SD) or N (%)) were calculated by gender; t-tests or chi-square tests were used to compare genders. Separate linear regression models were used to examine the relations of physical activity and sedentary behavior (assessed using accelerometry and CHAMPS) with BMI and grip strength. As previous studies have found sex differences in associations with BMI or grip strength,[20,40] models included sex as a covariate and a formal test of the interaction with sex was performed. Where sex differences were found (*P*<.05 for interaction term), the relations were estimated separately in men and women. In addition to the model adjusting only for sex, we also adjusted for other potential confounders. Finally, we examined whether the associations between physical activity (and sedentary behavior) and grip strength differed by BMI group (<30 kg/m^2^ vs. > = 30) by including physical activity and BMI group interaction terms. No explicit adjustment for multiplicity was made in these analyses.

## Results

### Descriptive statistics

BMI was not significantly different between men and women, while men had stronger grip than women (Table 1). For both self-reported and accelerometry-based physical activity measures, men and women spent the majority of time sedentary, followed by time in lower and then higher-light intensity physical activity. As expected, a small fraction of time was spent in moderate to vigorous intensity activity (approximately 4 minutes/day in men, and 2.5 minutes/day in women when assessed via accelerometry). Women spent more time sedentary than men, and less time physically active. The most prevalent activity types in each intensity category were watching television (sedentary), visiting with friends (lower-light intensity activity), and undertaking light house work (higher-light intensity activity; shown in S1 Table).

**Table 1:** Descriptive statistics of the Lifestyle Interventions and Independence for Elders (LIFE) Study sample at baseline, by sex (n = 1130).

	Men	Women	P*
Age (years)	79.3 (5.3)	78.5 (5.3)	.008
Race (% non-white)	56 (13.4)	243 (28.7)	<.001
Weight (kg)	91.3 (18.9)	77.7 (17.2)	<.001
Height (cm)	174.1 (7.3)	159.9 (6.8)	<.001
Body mass index (kg/m^2^)	30.1 (5.5)	30.3 (6.3)	.414
Grip strength (kg)	31.7 (10.2)	19.9 (6.3)	<.001
CHAMPS measures (self-report)			
Total physical activity (min/day)	125.6 (75.2)	143.4 (80.1)	<.001
Sedentary time (min/day)	197.5 (60.9)	184.6 (62.1)	<.001
Lower-light intensity (min/day)	49.1 (39.2)	63.3 (45.1)	<.001
Higher-light intensity (min/day)	67.9 (51.9)	74.1 (53.9)	.050
Accelerometry measures			
Total physical activity (min /day)	168.7 (67.0)	202.0 (67.9)	<.001
Sedentary time (min/day)	663.1 (109.6)	634.3 (114.7)	<.001
Lower-light intensity (min/day)	152.6 (55.7)	187.5 (59.0)	<.001
Higher-light intensity (min/day)	12.1 (13.1)	12.1 (11.6)	.950

Note: Results are presented as mean (SD) or as n (%);

*Comparison of sexes using t-tests or chi-squared tests; all accelerometry measures were adjusted for wear time; accelerometer cut points were as follows: sedentary time <100 counts/min; lower-light intensity: 100–1040 counts/min; Higher-light intensity: ≥1041 counts/min.

### Physical activity and sedentary behavior in relation to BMI

Using accelerometry, higher total physical activity was associated with lower BMI (Table 2). Less time spent sedentary and greater time spent in both low and higher-light intensity activities were associated with lower BMI. The magnitude of these associations was generally modest: for example, in the fully adjusted model, a 1-hour increment in lower-light intensity activity was associated with a 0.46kg/m^2^ (~1.5%) lower BMI.

**Table 2:** Mean differences in body mass index (kg/m^2^) per unit increase in physical activity and sedentary time.

	Minimally adjusted*		Fully adjusted**		
	β (95% CI)	P	β (95% CI)	P	Sex Interaction P-value
Accelerometry measures (n = 1193)					
Total physical activity (hr/day)	-0.12(-0.19,-0.06)	<.001	-0.11(-0.17,-0.04)	.003	.196
Sedentary time (hr/day)	0.42(0.13,0.71)	.005	0.44(0.14,0.74)	.004	.822
Lower-light intensity (hr/day)	-0.40(-0.74,-0.06)	.020	-0.46(-0.81,-0.12)	.009	.052
Higher-light intensity (hr/day)	-1.61(-3.20,-0.02)	.047	-1.19(-2.80,0.42)	.147	.326
CHAMPS self-reported measures (n = 1193)					
Total physical activity (hr/day)	-0.65(-1.00,-0.30)	<.001	-0.59(-0.96,-0.23)	.001	.942
Sedentary time (hr/day)	0.37(0.06,0.67)	.018	0.51(0.19,0.82)	.002	.531
Lower-light intensity (hr/day)	0.51(0.08,0.95)	.019	0.52(0.08,0.96)	.021	.121
Higher-light intensity (hr/day)	-0.46(-0.81,-0.11)	.010	-0.40(-0.76,-0.03)	.032	.617

*Adjusted for age, sex, and total wear time in hours (accelerometer measures only);

**Additionally adjusted for race, alcohol intake, smoking, education, diabetes, clinical site, comorbidity, history of arthritis or rheumatism, self-reported health;

^a^sex-specific coefficients: β (95% CI): -2.16(-3.79,-0.54) in men, -1.11(-2.77,0.55) in women.

accelerometer cut points were as follows: sedentary time <100 counts/min; lower-light intensity: 100–1040 counts/min; Higher-light intensity: 1041–1951 counts/min.

Higher self-reported total physical activity was also associated with lower BMI. Less time spent sedentary and more time spent in higher-light intensity activity were associated with lower BMI. In contrast, more time spent in lower-light intensity activity was associated with higher BMI. The positive association between total sedentary time and BMI was driven by television viewing; reading and computer use were not associated with BMI (S2 Table).

These patterns of association were similar in minimally and fully adjusted models (Table 2), although the inverse association between objectively assessed higher-light intensity activity and BMI was weaker in fully adjusted models and 95% confidence intervals overlapped the null. While there was no evidence for sex differences in associations (p for interaction term >0.05), there was some evidence that the inverse association between objectively assessed lower-light intensity activity and BMI was stronger in men than women (p for interaction term = 0.052). Associations between these exposures and waist circumference were similar to those with BMI (S3 Table).

### Physical activity and sedentary behavior in relation to grip strength

Using accelerometry, total physical activity was differentially associated with grip strength in men and women (p interaction term = .006; Table 3), with no association found in women and a positive association found in men. Sedentary and lower-light intensity activity time were not associated with grip strength, while greater time spent in higher-light intensity activity was associated with greater grip strength in men but not women (p interaction term = 0.002): a 1 hour increment in higher-light intensity physical activity was associated with a 6kg (~19%) higher grip strength among men. This association was similar after adjustment for BMI and other potential confounders. There was also no evidence that the association differed across BMI groups (p>0.1 for all physical activity by BMI group interactions). Using self-reported information, total physical activity, low and higher-light intensity activity, and total sedentary time were not associated with grip strength (Table 3).

**Table 3:** Mean differences in grip strength (kg) per unit increase in physical activity and sedentary time.

	Minimally adjusted*		Fully adjusted**		
	β (95% CI)	P	β (95% CI)	P	Sex Interaction P-value
Accelerometry measures (n = 1130)					
Total physical activity (hr/day)	0.06(-0.03,0.16)	.191	0.09(-0.01,0.18)	.075	.006
Total physical activity, men	0.14(-0.05,0.33)	.138	0.21(0.02,0.41)	.035	
Total physical activity, women	0.01(-0.09,0.11)	.897	0.01(-0.08,0.11)	.767	
Sedentary time (hr/day)	-0.13(-0.55,0.28)	.527	-0.22(-0.64,0.19)	.290	.905
Lower-light intensity (hr/day)	0.06(-0.42,0.54)	.809	0.16(-0.32,0.64)	.516	.140
Higher-light intensity (hr/day)	2.41(0.16,4.66)	.036	2.49(0.28,4.71)	.027	.002
Higher-light intensity, men	5.53(0.89,10.16)	.020	6.05(1.44,10.65)	.010	
Higher-light intensity, women	0.50(-1.85,2.86)	.675	0.49(-1.78,2.77)	.669	
CHAMPS self-reported measures(n = 1130)					
Total physical activity (hr/day)	-0.12(-0.62,0.39)	.650	-0.29(-0.80,0.21)	0.255	0.215
Sedentary time (hr/day)	0.10(-0.33,0.53)	.640	0.17(-0.27,0.60)	0.445	0.224
Lower-light intensity (hr/day)	0.02(-0.60,0.63)	.958	0.01(-0.60,0.62)	0.983	0.333
Higher-light intensity (hr/day)	-0.26(-0.76,0.23)	.298	-0.39(-0.88,0.11)	0.127	0.180

*adjusted for age, sex, total wear time in hours (accelerometer measures only).

**additionally adjusted for height, body mass index, race, alcohol intake, smoking, education, diabetes, clinical site, comorbitiy, history of arthritis or rheumatism, self-rated health.

accelerometer cut points were as follows: sedentary time <100 counts/min; lower-light intensity: 100–1040 counts/min; Higher-light intensity: 1041–1951 counts/min.

## Discussion

### Main findings

In cross-sectional analysis of relatively sedentary older adults at heightened risk of mobility disability, higher-light intensity physical activity and less sedentary time were associated with lower BMI. These associations were found using both self-reported and objectively assessed measures of physical activity and sedentary time. In contrast, greater time spent in higher-light activity (assessed objectively) was associated with greater grip strength in men (independent of BMI) but not women, while sedentary time was not associated with grip strength.

These results add to previous studies examining how self-reported physical activity and sedentary time relate to BMI (or weight) and grip strength in older adults.[15–17,20] Results from this study extend previous findings by using both objective and self-reported measures, and by explicitly investigating light intensity activity.

### Explanation of results

BMI is highly correlated with directly measured fat mass in older adults (e.g., r = 0.7[41]), and so associations with BMI are likely to be driven by fat mass. In support of this, patterns of association with waist circumference (which excludes appendicular muscle mass) were similar. The association between higher physical activity and lower BMI is consistent with the theory that fat mass levels are governed by energy balance: even light intensities of physical activity may contribute to lower energy balance, and in turn to lower BMI. Consistent with this, when using accelerometry, the magnitude of association was larger for higher-light than lower-light intensity activity. Associations with total and higher-light activity were found using both objective and self-reported activity, suggesting that both ranked participants similarly according to activity level. However, lower-light intensity activity was inversely associated with BMI when using accelerometry but not self-reported activity. This may be explained by the imprecise classification of self-reported activity intensities—those grouped as lower-light intensity activities in CHAMPS include potentially sedentary components (e.g., “visiting friends or family”), which could lead to misclassification. This is supported by the positive correlation between sedentary and lower-light time (partial r (as per Table 2) = .21) and a study which found that lower-light intensity activity derived from CHAMPs was only weakly related to a comparable accelerometry-assessed measure.[39]

The association between greater sedentary time and higher BMI is likely to be partly or wholly explained by more sedentary participants spending less time in light intensity activity—such that each hour increase in time spent sedentary reflects an hour decrease in time spent physically active. Sedentary time was inversely correlated with total physical activity (partial r (as per Table 2) = -0.88). Because of this, when using accelerometry data we were unable to determine whether sedentary time was associated with outcomes independent of physical activity time, due to the occurrence of multicollinearity in fully adjusted models. If the detrimental effects of food-related television advertisements on obesity in children[42] extend to older adults, the positive associations between sedentary time and BMI could also be explained, at least in part, by dietary factors.

The magnitude of associations between physical activity and BMI were generally similar in men and women. While it has been suggested that women may be less responsive to the effects of physical activity on weight,[43,44] recent experimental studies in young adults, which have either measured or controlled for energy expenditure, have found no sex differences in weight loss response to exercise.[45,46]

Associations between higher physical activity and stronger grip were only found for higher-light intensity activity when measured by accelerometry in men. It is possible that men may have more frequently taken part in the types of activities that benefited upper extremity muscle strength than women, as suggested in other studies.[40] Given the sedentary nature of the cohort, these activities are unlikely to be resistance exercises,[13] but rather domestic and leisure activities that result in both load bearing of the muscle and movement along the vertical axis. Consistent with this, aerobically active adults have been found to have greater muscle strength than their less active age-matched counter-parts,[47] and a number of experimental studies have found that aerobic exercise training can lead to skeletal muscle hypertrophy.[48] The lack of association between self-reported physical activity and grip strength could suggest that the items used did not fully capture the types of activities affecting muscle strength or that the activities leading to greater muscle strength were not undertaken by participants of this study. The larger random measurement error expected in self-reported activity would also drive the associations towards the null.[49]

### Strengths and limitations

Strengths of this study include the use of a large sample of older men and women at heightened risk of mobility disability that have been rarely studied, and the inclusion of both self-reported and objectively assessed measures of light intensity physical activity and sedentary time. The self-reported measures captured information on the types of activities undertaken, while the objective measures may have provided more accurate assessment of light intensity physical activity and sedentary time volumes.

Limitations of the current study include the cross-sectional observational design, which provides limited evidence with which to make causal inferences. While we hypothesized that physical activity and sedentary behavior would be associated with BMI and grip strength, associations are likely to be bi-directional in nature, as high BMI and low muscle strength may lead to lower physical activity participation. The sedentary nature of the LIFE cohort precluded the investigation of moderate to vigorous intensity physical activity, although there was sufficient variation in activity levels to investigate associations with time spent sedentary and in light intensity of activity. The use of BMI as an indicator of fat mass may have led us to underestimate the associations with physical activity, as physical activity may be positively associated with lean mass.[14,50] The accelerometer did not distinguish between different postures (i.e., standing, sitting, or lying), and time spent in each could impact differently on fat mass and muscle strength. While a recent consensus definition of sedentary behavior specifies that it is behavior undertaken while sitting,[38] laying down or reclining also result in low energy expenditure,[51] and daytime lying (e.g., napping or reclining) is a prevalent form of sedentary behavior among older adults.[52]

### Implications

In this cross-sectional study, greater time spent in light intensity activity and lower sedentary time were both associated with lower BMI. These findings are consistent with the hypothesis that replacing some of the highly prevalent sedentary time[30] with light intensity physical activity might lead to lower BMI levels and therefore obesity prevalence among older adults; however, longitudinal and experimental studies are needed to strengthen causal inferences. Our findings support future intervention studies designed to reduce television viewing time to reduce BMI, replacing television viewing with light intensity activities such as leisurely walking. Further studies could also provide additional information on the benefits of different types of activity: activities which benefit both fat mass and muscle strength may be particularly beneficial, given their independent or synergistic effects on health and physical functioning. We found, for example, that greater time spent in higher-light intensity activities was associated with both lower BMI and stronger grip strength in men, and that the association with grip strength was independent of and not modified by BMI. This suggests that this intensity of activity was associated with lower risk of obesity and low grip strength (ie, dynapenic obesity)—however, other cohorts may be more suited to examine associations specifically with dynapenic obesity, as most participants in the LIFE cohort were obese according to BMI. Given logistical difficulties in recruiting older adults into behavioral physical activity interventions, and low rates, in general, of long-term post-intervention improvements in physical activity,[53] multiple interventions may be required at both the individual and societal levels to increase population physical activity levels.

The results from this study also support the use of self-reported measures of sedentary behavior and physical activity in older adults, given their correlation with BMI, a finding that was also observed using objective measures. Further work is needed to derive valid and reliable measures of lower-light intensity activity among older adults.[39]

### Conclusions

In a sample of sedentary older adults at risk of mobility disability, higher-light intensity physical activity was cross-sectionally associated with lower BMI, and more time spent sedentary was associated with higher BMI. In contrast, higher-light intensity activity was differentially associated with grip strength in men and women (positively associated among men when assessed using accelerometry only), while self-reported measures were not associated with grip strength. These results are consistent with the hypothesis that replacing highly prevalent sedentary activities, especially television viewing, with light intensity physical activities such as leisurely walking could lead to lower BMI levels and therefore obesity prevalence among the population of older adults. Further longitudinal and experimental studies are required to confirm these findings and identify interventions to reduce BMI and improve grip strength.

## Supporting Information

S1 Appendix Questionnaire items used to classify time spent per week sedentary, or in low- to higher-light intensity physical activity from the shortened version of the Community Health Activities Model Program for Seniors physical activity questionnaire (CHAMPS). (DOCX)

S2 Appendix Research Investigators for the LIFE Study. (DOCX)

S1 Table Frequency of participation in sedentary behaviors and physical activities assessed using the Community Health Activities Model Program for Seniors physical activity questionnaire. (DOCX)

S2 Table Mean differences in body mass index (kg/m^2^) per hour/day increase in self-reported sedentary behaviors (n = 1193). (DOCX)

S3 Table Mean differences in waist circumference (cm) per unit increase in physical activity and sedentary time. (DOCX)
