# Correction: Light Intensity Physical Activity and Sedentary Behavior in Relation to Body Mass Index and Grip Strength in Older Adults: Cross-Sectional Findings from the Lifestyle Interventions and Independence for Elders (LIFE) Study

**DOI:** 10.1371/journal.pone.0126063

**Published:** 2015-04-15

**Authors:** David Bann, Don Hire, Todd Manini, Rachel Cooper, Anda Botoseneanu, Mary M. McDermott, Marco Pahor, Nancy W. Glynn, Roger Fielding, Abby C. King, Timothy Church, Walter T. Ambrosius, Thomas M. Gill

The thirteenth author’s name is spelled incorrectly. The correct name is: Thomas M. Gill.
