# Supplementary material for: Light Intensity Physical Activity and Sedentary Behavior in Relation to Body Mass Index and Grip Strength in Older Adults: Cross-Sectional Findings from the Lifestyle Interventions and Independence for Elders (LIFE) Study
Source: PLoS One. 2015 Feb 3;10(2):e0116058. doi: 10.1371/journal.pone.0116058 (PMC4315494; doi:10.1371/journal.pone.0116058)
Supplement: S1 Appendix — (DOCX) [file pone.0116058.s001.docx]

Appendix S1. Questionnaire items used to classify time spent per week sedentary, or in low- to higher-light intensity physical activity from the shortened version of the Community Health Activities Model Program for Seniors physical activity questionnaire (CHAMPS).

Sedentary (≤1.5 METs): Watching television; using a computer; reading; attend a concert, movie, lecture, or sport event; play cards, bingo, or board games with other people; attend other club or group meetings.

Lower-light intensity activities (>1.5 to 2 METs): Visit with friends or family (other than those you live with); Go to senior center; attend church or take part in church activities; do woodworking, needlework, drawing, or other arts and crafts; play a musical instrument.

Higher-light intensity activities (>2 to <3 METs): Do volunteer work; Play golf, riding a cart (count walking time only); shoot pool or billiards; Do light work around the house (such as sweeping or vacuuming); do light gardening (such as watering plants); walk to do errands [such as to/from a store or to take children to school (count walk time only)]; walk leisurely for exercise or pleasure; do stretching or flexibility exercises (do not count yoga or Tai-chi); do yoga or Tai-chi; do general conditioning exercises, such as light calisthenics or chair exercises (do not count time on sidelines).

Moderate to vigorous intensity activities (≥3 METs): Dance (such as square, fold, line ballroom)(do not count aerobic dance here); Play golf, carrying or pulling your equipment (count walking time only); singles or doubles tennis; Skate (ice, roller, in-line); Do heavy work around the house (such as washing windows, cleaning gutters) ; Do heavy gardening (such as spading, raking); Jog or run ; Walk uphill or hike uphill (count only uphill part); Walk fast or briskly for exercise (do not count walking leisurely or uphill); Ride a bicycle or stationary cycle; Do other aerobic machines such as rowing, or step machines (do not count treadmill or stationary cycle); Do water exercises (do not count other swimming); Swim moderately or fast; Swim gently; Do aerobics or aerobic dancing; Do moderate to heavy strength training (such as hand-held weights of more than 5 lbs., weight machines or push-ups); Do light strength training (such as hand-held weights of 5 lbs. or less or elastic bands); Play basketball, soccer, or racquetball (do not count time on sidelines).

Total physical activity: time spent in lower-light + higher-light + moderate to vigorous intensity activity
