# Supplementary material for: Light Intensity Physical Activity and Sedentary Behavior in Relation to Body Mass Index and Grip Strength in Older Adults: Cross-Sectional Findings from the Lifestyle Interventions and Independence for Elders (LIFE) Study
Source: PLoS One. 2015 Feb 3;10(2):e0116058. doi: 10.1371/journal.pone.0116058 (PMC4315494; doi:10.1371/journal.pone.0116058)
Supplement: S2 Appendix — (DOCX) [file pone.0116058.s002.docx]

Appendix S2. Research Investigators for the LIFE Study.

The Lifestyle Interventions and Independence for Elders Study is funded by a National Institutes of Health/National Institute on Aging Cooperative Agreement #UO1 AG22376 and a supplement from the National Heart, Lung and Blood Institute 3U01AG022376-05A2S, and sponsored in part by the Intramural Research Program, National Institute on Aging, NIH.

The research is partially supported by the Claude D. Pepper Older Americans Independence Centers at the University of Florida (1 P30 AG028740), Wake Forest University (1 P30 AG21332),Tufts University (1P30AG031679), University of Pittsburgh (P30 AG024827), and Yale University (P30AG021342) and the NIH/NCRR CTSA at Stanford University (UL1 RR025744),

Tufts University is also supported by the Boston Rehabilitation Outcomes Center (1R24HD065688-01A1).

LIFE investigators are also partially supported by the following:

Dr. Thomas Gill (Yale University) is the recipient of an Academic Leadership Award (K07AG3587) from the National Institute on Aging.

Dr. Carlos Fragoso (Spirometry Reading Center, Yale University) is the recipient of a Career Development Award from the Department of Veterans Affairs.

Dr. Roger Fielding (Tufts University) is partially supported by the U.S. Department of Agriculture, under agreement No. 58-1950-0-014. Any opinions, findings, conclusion, or recommendations expressed in this publication are those of the author(s) and do not necessarily reflect the view of the U.S. Dept of Agriculture.

**Administrative Coordinating Center, University of Florida, Gainesville, FL**

Marco Pahor, MD – Principal Investigator of the LIFE Study

Jack M. Guralnik, MD, PhD – Co-Investigator of the LIFE Study (University of Maryland School of Medicine, Baltimore, MD)

Christiaan Leeuwenburgh, PhD

Connie Caudle

Lauren Crump, MPH

Latonia Holmes

Jocelyn Lee, PhD

Ching-ju Lu, MPH

**Data Management, Analysis and Quality Control Center, Wake Forest University, Winston Salem, NC**

Michael E. Miller, PhD – DMAQC Principal Investigator

Mark A. Espeland, PhD – DMAQC Co-Investigator

Walter T. Ambrosius, PhD

William Applegate, MD

Daniel P. Beavers, PhD, MS

Robert P. Byington, PhD, MPH, FAHA

Delilah Cook, CCRP

Curt D. Furberg, MD, PhD

Lea N. Harvin, BS

Leora Henkin, MPH, Med

John Hepler, MA

Fang-Chi Hsu, PhD

Laura Lovato, MS

Wesley Roberson, BSBA

Julia Rushing, BSPH, MStat

Scott Rushing, BS

Cynthia L. Stowe, MPM

Michael P. Walkup, MS

Don Hire, BS

W. Jack Rejeski, PhD

Jeffrey A. Katula, PhD, MA

Peter H. Brubaker, PhD

Shannon L. Mihalko, PhD

Janine M. Jennings, PhD

**National Institutes of Health, Bethesda, MD**

Evan C. Hadley, MD (National Institute on Aging)

Sergei Romashkan, MD, PhD (National Institute on Aging)

Kushang V. Patel, PhD (National Institute on Aging)

**National Heart, Lung and Blood Institute, Bethesda, MD**

Denise Bonds, MD, MPH

**Field Centers**

**Northwestern University, Chicago, IL**

Mary M. McDermott, MD – Field Center Principal Investigator

Bonnie Spring, PhD – Field Center Co-Investigator

Joshua Hauser, MD – Field Center Co-Investigator

Diana Kerwin, MD – Field Center Co-Investigator

Kathryn Domanchuk, BS

Rex Graff, MS

Alvito Rego, MA

**Pennington Biomedical Research Center, Baton Rouge, LA**

Timothy S. Church, MD, PhD, MPH – Field Center Principal Investigator

Steven N. Blair, PED (University of South Carolina)

Valerie H. Myers, PhD

Ron Monce, PA-C

Nathan E. Britt, NP

Melissa Nauta Harris, BS

Ami Parks McGucken, MPA, BS

Ruben Rodarte, MBA, MS, BS

Heidi K. Millet, MPA, BS

Catrine Tudor-Locke, PhD, FACSM

Ben P. Butitta, BS

Sheletta G. Donatto, MS, RD, LDN, CDE

Shannon H. Cocreham, BS

**Stanford University, Palo Alto, CA**

Abby C. King, PhD – Field Center Principal Investigator

Cynthia M. Castro, PhD
William L. Haskell, PhD

Randall S. Stafford, MD, PhD
Leslie A. Pruitt, PhD

Kathy Berra, MSN, NP-C, FAAN

Veronica Yank, MD

**Tufts University, Boston, MA**

Roger A. Fielding, PhD – Field Center Principal Investigator

Miriam E. Nelson, PhD – Field Center Co-Investigator

Sara C. Folta, PhD – Field Center Co-Investigator

Edward M. Phillips, MD

Christine K. Liu, MD

Erica C. McDavitt, MS

Kieran F. Reid, PhD, MPH

Dylan R. Kirn, BS

Evan P. Pasha, BS

Won S. Kim, BS

Vince E. Beard, BS

Eleni X. Tsiroyannis, BS

Cynthia Hau, BS, MPH

**University of Florida, Gainesville, FL**

Todd M. Manini, PhD – Field Center Principal Investigator

Marco Pahor, MD – Field Center Co-Investigator

Stephen D. Anton, PhD

Susan Nayfield, MD

Thomas W. Buford, PhD

Michael Marsiske, PhD

Bhanuprasad D. Sandesara, MD

Jeffrey D. Knaggs, BS

Megan S. Lorow, BS

William C. Marena, MT, CCRC

Irina Korytov, MD

Holly L. Morris, MSN, RN, CCRC (Brooks Rehabilitation Clinical Research Center, Jacksonville, FL)

Margo Fitch, PT (Brooks Rehabilitation Clinical Research Center, Jacksonville, FL)

Floris F. Singletary, MS, CCC-SLP (Brooks Rehabilitation Clinical Research Center, Jacksonville, FL)

Jackie Causer, BSH, RN (Brooks Rehabilitation Clinical Research Center, Jacksonville, FL)

Katie A. Radcliff, MA (Brooks Rehabilitation Clinical Research Center, Jacksonville, FL)

**University of Pittsburgh, Pittsburgh, PA**

Anne B. Newman, MD, MPH – Field Center Principal Investigator

Stephanie A. Studenski, MD, MPH – Field Center Co-Investigator

Bret H. Goodpaster, PhD

Nancy W. Glynn, PhD

Oscar Lopez, MD

Neelesh K. Nadkarni, MD, PhD

Kathy Williams, RN, BSEd, MHSA

Mark A. Newman, PhD

George Grove, MS

Janet T. Bonk, MPH, RN

Jennifer Rush, MPH

Piera Kost, BA (deceased)

Diane G. Ives, MPH

**Wake Forest University, Winston Salem, NC**

Stephen B. Kritchevsky, Ph.D. – Field Center Principal Investigator

Anthony P. Marsh, PhD – Field Center Co-Investigator

Tina E. Brinkley, PhD

Jamehl S. Demons, MD

Kaycee M. Sink, MD, MAS

Kimberly Kennedy, BA, CCRC

Rachel Shertzer-Skinner, MA, CCRC

Abbie Wrights, MS

Rose Fries, RN, CCRC

Deborah Barr, MA, RHEd, CHES

**Yale University, New Haven, CT**

Thomas M. Gill, MD – Field Center Principal Investigator

Robert S. Axtell, PhD, FACSM – Field Center Co-Investigator (Southern Connecticut State University, Exercise Science Department)

Susan S. Kashaf, MD, MPH (VA Connecticut Healthcare System)

Nathalie de Rekeneire, MD, MS

Joanne M. McGloin, MDiv, MS, MBA

Karen C. Wu, RN

Denise M. Shepard, RN, MBA

Barbara Fennelly, MA, RN

Lynne P. Iannone, MS, CCRP

Raeleen Mautner, PhD

Theresa Sweeney Barnett, MS, APRN

Sean N. Halpin, MA

Matthew J. Brennan, MA

Julie A. Bugaj, MS

Maria A. Zenoni, MS

Bridget M. Mignosa, AS

**Cognition Coordinating Center, Wake Forest University, Winston Salem, NC**

Jeff Williamson, MD, MHS – Center Principal Investigator

Kaycee M Sink, MD, MAS – Center Co-Investigator

Hugh C. Hendrie, MB, ChB, DSc (Indiana University)

Stephen R. Rapp, PhD

Joe Verghese, MB, BS (Albert Einstein College of Medicine of Yeshiva University)

Nancy Woolard

Mark Espeland, PhD

Janine Jennings, PhD

**Electrocardiogram Reading Center, University of Florida, Gainesville, FL**

Carl J. Pepine MD, MACC

Mario Ariet, PhD

Eileen Handberg, PhD, ARNP

Daniel Deluca, BS

James Hill, MD, MS, FACC

Anita Szady, MD

**Spirometry Reading Center, Yale University, New Haven, CT**

Geoffrey L. Chupp, MD

Gail M. Flynn, RCP, CRFT

Thomas M. Gill, MD

John L. Hankinson, PhD (Hankinson Consulting, Inc.)

Carlos A. Vaz Fragoso, MD

**Cost Effectiveness Analysis Center**

Erik J. Groessl, PhD (University of California, San Diego and VA San Diego Healthcare System)

Robert M. Kaplan, PhD (Office of Behavioral and Social Sciences Research, National Institutes of Health)
