# Supplementary material for: Light Intensity Physical Activity and Sedentary Behavior in Relation to Body Mass Index and Grip Strength in Older Adults: Cross-Sectional Findings from the Lifestyle Interventions and Independence for Elders (LIFE) Study
Source: PLoS One. 2015 Feb 3;10(2):e0116058. doi: 10.1371/journal.pone.0116058 (PMC4315494; doi:10.1371/journal.pone.0116058)
Supplement: S1 Table — (DOCX) [file pone.0116058.s003.docx]

Table S1. Frequency of participation in sedentary behaviors and physical activities assessed using the Community Health Activities Model Program for Seniors physical activity questionnaire.

| Category | Activity | Mean  min/week (SD) | Median  min/week | 25^th^- 75^th^ centile | % contribution  to category |
| --- | --- | --- | --- | --- | --- |
| 1-Sedentary | Watch television | 511.9 (150.0) | 585.0 | 465.0-585.0 | 38.7 |
|  | Read | 431.3 (183.9) | 465.0 | 225.0-585.0 | 32.6 |
|  | Use computer | 247.5 (252.3) | 105.0 | 0.0-585.0 | 18.7 |
|  | Play cards, board games | 46.6 (117.9) | 0.0 | 0.0-0.0 | 3.5 |
|  | Attend other club | 43.6 (100.8) | 0.0 | 0.0-0.0 | 3.3 |
|  | Attend concert, sport | 43.1 (102.5) | 0.0 | 0.0-0.0 | 3.3 |
|  | Total Sedentary Minutes | 1322.0 (434.0) | 1275.0 | 1035.0-1635.0 |  |
| 2-Low-light | Visit with friends | 215.1 (193.5) | 225.0 | 30.0-345.0 | 52.4 |
|  | Attend church | 109.4 (130.6) | 105.0 | 0.0-225.0 | 26.6 |
|  | Woodworking, crafts | 54.1 (144.7) | 0.0 | 0.0-0.0 | 13.2 |
|  | Go to senior center | 20.3 (82.2) | 0.0 | 0.0-0.0 | 5.0 |
|  | Play musical instrument | 11.7 (56.9) | 0.0 | 0.0-0.0 | 2.8 |
|  | Total lower-light minutes | 410.6 (306.0) | 330.0 | 210.0-585.0 |  |
| 3-High-light and above | Do light house work | 195.5 (186.3) | 105.0 | 30.0-345.0 | 35.4 |
|  | Walk leisurely | 71.1 (126.9) | 0.0 | 0.0-105.0 | 12.9 |
|  | Do light gardening | 68.5 (115.1) | 30.0 | 0.0-105.0 | 12.4 |
|  | Do volunteer work | 63.6 (140.9) | 0.0 | 0.0-0.0 | 11.5 |
|  | Walk to do errands | 51.6 (111.7) | 0.0 | 0.0-30.0 | 9.3 |
|  | Do stretching exercises | 36.9 (75.6) | 0.0 | 0.0-30.0 | 6.7 |
|  | Do heavy gardening | 18.6 (79.6) | 0.0 | 0.0-0.0 | 3.4 |
|  | Do heavy house work | 11.9 (65.9) | 0.0 | 0.0-0.0 | 2.2 |
|  | Do general conditioning | 10.6 (37.3) | 0.0 | 0.0-0.0 | 1.9 |
|  | Do light strength training | 4.9 (18.1) | 0.0 | 0.0-0.0 | 0.9 |
|  | Golf (ride cart) | 3.3 (33.3) | 0.0 | 0.0-0.0 | 0.6 |
|  | Walk fast | 2.9 (15.3) | 0.0 | 0.0-0.0 | 0.5 |
|  | Do yoga | 2.8 (23.0) | 0.0 | 0.0-0.0 | 0.5 |
|  | Ride bicycle | 2.6 (13.8) | 0.0 | 0.0-0.0 | 0.5 |
|  | Walk uphill | 1.7 (9.6) | 0.0 | 0.0-0.0 | 0.3 |
|  | Do water exercises | 1.6 (12.3) | 0.0 | 0.0-0.0 | 0.3 |
|  | Shoot pool | 1.2 (16.5) | 0.0 | 0.0-0.0 | 0.2 |
|  | Work on car, machinery | 1.0 (9.6) | 0.0 | 0.0-0.0 | 0.2 |
|  | Dance | 0.9 (8.7) | 0.0 | 0.0-0.0 | 0.2 |
|  | Do heavy strength training | 0.8 (6.9) | 0.0 | 0.0-0.0 | 0.1 |
|  | Swim gently | 0.3 (4.0) | 0.0 | 0.0-0.0 | 0.1 |
|  | Do aerobic machines | 0.2 (2.7) | 0.0 | 0.0-0.0 | 0.0 |
|  | Play doubles tennis | 0.2 (4.2) | 0.0 | 0.0-0.0 | 0.0 |
|  | Swim moderately | 0.1 (3.1) | 0.0 | 0.0-0.0 | 0.0 |
|  | Golf (carry clubs) | 0.1 (3.0) | 0.0 | 0.0-0.0 | 0.0 |
|  | Jog or run | 0.0 (1.2) | 0.0 | 0.0-0.0 | 0.0 |
|  | Play basketball | 0.0 (0.8) | 0.0 | 0.0-0.0 | 0.0 |
|  | Play singles tennis | 0.0 (0.8) | 0.0 | 0.0-0.0 | 0.0 |
|  | Do aerobics | 0.0 (0.0) | 0.0 | 0.0-0.0 | 0.0 |
|  | Skate | 0.0 (0.0) | 0.0 | 0.0-0.0 | 0.0 |
|  | Total higher-light and above minutes | 552.8 (403.1) | 480.0 | 240.0-795.0 |  |
|  |  |  |  |  |  |
|  |  |  |  |  |  |
