# Supplementary material for: Light Intensity Physical Activity and Sedentary Behavior in Relation to Body Mass Index and Grip Strength in Older Adults: Cross-Sectional Findings from the Lifestyle Interventions and Independence for Elders (LIFE) Study
Source: PLoS One. 2015 Feb 3;10(2):e0116058. doi: 10.1371/journal.pone.0116058 (PMC4315494; doi:10.1371/journal.pone.0116058)
Supplement: S2 Table — (DOCX) [file pone.0116058.s004.docx]

Table S2. Mean differences in body mass index (kg/m^2^) per hour/day increase in self-reported sedentary behaviors (n=1193).

|  | | | | | | Minimally adjusted* |  | Fully adjusted** | |  | |  | |  |  |  |  |  |  |  |  |  |  |
| --- | --- | --- | --- | --- | --- | --- | --- | --- | --- | --- | --- | --- | --- | --- | --- | --- | --- | --- | --- | --- | --- | --- | --- |
|  | | | | | | Β (95% CI) | P | Β (95% CI) | | P | | P# | |  |  |  |  |  |  |  |  |  |  |
|  |  |  |  |  |  |  |  |  |  |  |  |  |  |  |  |  |  |  |  |  |  |  |  |
| Watching television (hr/day) | | | | | | 1.71(0.84,2.58) | <.001 | 1.48(0.60,2.37) | | .001 | | .184 | |  |  |  |  |  |  |  |  |  |  |
| Reading (hr/day) | | | | | | -0.45(-1.16,0.27) | .220 | -0.04(-0.78,0.70) | | .913 | | .714 | |  |  |  |  |  |  |  |  |  |  |
| Using a computer (hr/day) | | | | | | 0.23(-0.30,0.76) | .403 | 0.35(-0.20,0.90) | | .217 | | .164 | |  |  |  |  |  |  |  |  |  |  |
|  | | | | | |  |  |  |  | |  | |  | | | | | |  |  |  |  |  |

*adjusted for age, sex, total wear time in hours (accelerometer measures only).

**additionally adjusted for race, alcohol intake, smoking, education, diabetes, clinical site, comorbitiy, self-rated health.

accelerometer cut points were as follows: sedentary time <100 counts/min; lower-light intensity: 100-1040 counts/min; Higher-light intensity: 1041–1951 counts/min.
