# Supplementary material for: Light Intensity Physical Activity and Sedentary Behavior in Relation to Body Mass Index and Grip Strength in Older Adults: Cross-Sectional Findings from the Lifestyle Interventions and Independence for Elders (LIFE) Study
Source: PLoS One. 2015 Feb 3;10(2):e0116058. doi: 10.1371/journal.pone.0116058 (PMC4315494; doi:10.1371/journal.pone.0116058)
Supplement: S3 Table — (DOCX) [file pone.0116058.s005.docx]

Table S3. Mean differences in waist circumference (cm) per unit increase in physical activity and sedentary time.

|  | Minimally adjusted* |  | Fully adjusted** |  |  |  |
| --- | --- | --- | --- | --- | --- | --- |
|  | Β (95% CI) | P | Β (95% CI) | P | P# |  |
| Accelerometry measures (n=1130) |  |  |  |  |  |  |
| Total physical activity (hr/day) | -0.32(-0.48,-0.16) | <.001 | -0.27(-0.43,-0.10) | .002 | .293 |  |
| Sedentary time (hr/day) | 1.16(0.44,1.88) | .002 | 1.08(0.35,1.82) | .004 | .629 |  |
| Lower-light intensity (hr/day) | -1.14(-1.98,-0.31) | .007 | -1.14(-2.00,-0.28) | .009 | .088 |  |
| Higher-light intensity (hr/day) | -4.29(-8.19,-0.39) | .031 | -3.35(-7.29,0.60) | .096 | .361 |  |
|  |  |  |  |  |  |  |
| CHAMPS self-reported measures (n=1193) |  |  |  |  |  |  |
| Total physical activity (hr/day) | -1.11(-1.98,-0.23) | .013 | -1.00(-1.90,-0.10) | .030 | .506 |  |
| Sedentary time (hr/day) | 0.68(-0.07,1.43) | .077 | 0.85(0.08,1.63) | .032 | .409 |  |
| Lower-light intensity (hr/day) | 0.91(-0.16,1.98) | .095 | 0.93(-0.16,2.01) | .093 | .074 |  |
| Higher-light intensity (hr/day) | -0.91(-1.78,-0.05) | .039 | -0.77(-1.65,0.12) | .090 | .212 |  |
|  |  |  |  |  |  |  |

*adjusted for age, sex, total wear time in hours (accelerometer measures only).

**additionally adjusted for height, body mass index, race, alcohol intake, smoking, education, diabetes, clinical site, comorbitiy, history of arthritis or rheumatism, self-rated health.

accelerometer cut points were as follows: sedentary time <100 counts/min; lower-light intensity: 100-1040 counts/min; Higher-light intensity: 1041–1951 counts/min.
